# Radiation dose of digital radiography (DR) versus micro-dose x-ray (EOS) on patients with adolescent idiopathic scoliosis: 2016 SOSORT- IRSSD “John Sevastic Award” Winner in Imaging Research

**DOI:** 10.1186/s13013-016-0106-7

**Published:** 2016-12-29

**Authors:** Steve C. N. Hui, Jean-Philippe Pialasse, Judy Y. H. Wong, Tsz-ping Lam, Bobby K. W. Ng, Jack C. Y. Cheng, Winnie C. W. Chu

**Affiliations:** 1 Department of Imaging and Interventional Radiology, Prince of Wales Hospital, The Chinese University of Hong Kong, Sha Tin, Hong Kong, SAR China; 2 Department of Orthopedics & Traumatology, The Chinese University of Hong Kong, Sha Tin, Hong Kong, SAR China; 3 Department of Chiropractic, University of Quebec at Trois-Rivieres, Trois-Rivieres, Quebec Canada

**Keywords:** AIS, Radiation, Micro-dose 2D/3D slot-scanning x-ray, Entrance skin dose, Effective dose, Organ dose, Thermoluminescent dosimeters

## Abstract

**Background:**

Patients with adolescent idiopathic scoliosis (AIS) frequently receive x-ray imaging at diagnosis and subsequent follow monitoring. The ionizing radiation exposure has accumulated through their development stage and the effect of radiation to this young vulnerable group of patients is uncertain. To achieve the ALARA (as low as reasonably achievable) concept of radiation dose in medical imaging, a slot-scanning x-ray technique by the EOS system has been adopted and the radiation dose using micro-dose protocol was compared with the standard digital radiography on patients with AIS.

**Methods:**

Ninety-nine participants with AIS underwent micro-dose EOS and 33 underwent standard digital radiography (DR) for imaging of the whole spine. Entrance-skin dose was measured using thermoluminescent dosimeters (TLD) at three regions (i.e. dorsal sites at the level of sternal notch, nipple line, symphysis pubis). Effective dose and organ dose were calculated by simulation using PCXMC 2.0. Data from two x-ray systems were compared using independent-samples t-test and significance level at 0.05. All TLD measurements were conducted on PA projection only. Image quality was also assessed by two raters using Cobb angle measurement and a set of imaging parameters for optimization purposes.

**Results:**

Entrance-skin dose from micro-dose EOS system was 5.9–27.0 times lower at various regions compared with standard DR. The calculated effective dose was 2.6 ± 0.5 (μSv) and 67.5 ± 23.3 (μSv) from micro-dose and standard DR, respectively. The reduction in the micro-dose was approximately 26 times. Organ doses at thyroid, lung and gonad regions were significantly lower in micro-dose (*p* < 0.001). Data were further compared within the different gender groups. Females received significantly higher (*p* < 0.001) organ dose at ovaries compared to the testes in males. Patients with AIS received approximately 16–34 times lesser organ dose from micro-dose x-ray as compared with the standard DR. There was no significant difference in overall rating of imaging quality between EOS and DR. Micro-dose protocol provided enough quality to perform consistent measurement on Cobb angle.

**Conclusions:**

Entrance-skin dose, effective dose and organ dose were significantly reduced in micro-dose x-ray. The effective dose of a single micro-dose x-ray (2.6 μSv) was less than a day of background radiation. As AIS patients require periodic x-ray follow up for surveillance of curve progression, clinical use of micro-dose x-ray system is beneficial for these young patients to reduce the intake of ionizing radiation.

## Background

Patients with adolescent idiopathic scoliosis (AIS) suffer from 3-dimensional spinal deformities. The onset and progress occur during their youth stage and usually become stable after skeletal maturity. The current gold standard of diagnosis is made based on the measurement of a Cobb angle larger than 10°. As the chance of curve progression increases in younger patients with greater initial Cobb angle [1, 2], patients receive brace treatment and follow-up monitoring in routine basis at young age. During each follow-up, patients undergo digital radiography to capture images of spine which allow physicians to monitor their curve progression over time. As the treatment of AIS covers a relatively long period during their adolescence, the accumulation of ionizing radiation has become a concern for this vulnerable group of teenagers.

Ionizing radiation from x-ray has high enough energy to break molecular bonding in humans. Damaged bonding repaired incorrectly could affect chromosome to induce cancer [3, 4]. The accumulated ionizing radiation increases the probability of adverse health issues and uncertainties including cancer and abnormal pregnancy to patients. Retrospective studies indicated patients with AIS who frequently received x-ray have approximately 2 and 3% increased lifetime risk of breast cancer and heritable defect, respectively [5–7], and higher risks of unsuccessful attempts at pregnancy, spontaneous abortions, infants with congenital malformations and lower birthweight [8]. As pediatric patients have a longer lifetime to manifest radiation damage than adults and the adverse effects could appear years after exposure, it is important to call for special attention in radiation protection and apply any available methods to achieve the principle of ALARA (as low as reasonably achievable) to minimize the radiation.

Considerable efforts and improvements have been made to reduce the radiation dose from x-ray imaging. Increasingly, conventional film based radiographies are being replaced by the digital ones over the last 15 years. Moreover, the digital technique reduces the number of x-ray retake from 5.5% for conventional to 1.0%, which significantly avoids repeated exposure [9]. Literatures also suggest that the change of image orientation from anterior-posterior (AP) view to posterior-anterior (PA) view could greatly reduce the organ dose by approximately three- to eight-fold to the breasts and thyroid because of the lower sensitive organ dose to the anterior structures; thereby, it is suggested for routine spine examinations [10, 11]. Other technical improvement has been made to reduce the exposure including the use of 3-phase x-ray machines and high-speed x-ray films [12]. A new implementation of radiography, the slot-scanning x-ray by EOS 2D/3D system (EOS Imaging, Paris, France), has been adopted recently and it has a great advantage in capturing x-ray images using very low radiation dose. The EOS system is equipped with two sets of x-ray tube mounted at right angles, a biplanar design, and utilizes the multi-wire proportional chamber (MWPC) to detect charged particles and photons for simultaneous acquisition of frontal and lateral images [13]. The application of EOS mainly involves clinical measurement and analysis of spinal curvature in AIS [14–16], bone fracture [17], torsion [18–21], orientation and alignment of spine and lower body limb [22–25]. The accuracy, reliability and reproducibility of curve measurement using 3D reconstruction feature in EOS have also been tested and the results are comparable with manual 2D method and CT data [26, 27].

An early experimental study indicated that the entrance skin dose from slot-scanning x-ray technique using MWPC detector was reduced by 13 times at PA orientation and 15 times at lateral in a full spine procedure compared to the conventional film based radiography while no significant loss of diagnostic information [28]. The current EOS system also embedded with MWPC provides two strengths of acquisition protocols e.g. the standard low-dose and the micro-dose. Previous literature reported the entrance skin dose, using low-dose protocol, was reduced by 6 to 9 times with improved image quality compared with computed radiography [29]. A phantom based radiological study reported the effective dose of a full spine examination using EOS low-dose protocol was 290 μSv for an adult and 200 μSv for a child [30].

As micro-dose is a relatively new protocol from EOS, very limited number of publications is available regarding the radiation dose and image quality. To the best of our knowledge, a recent study reported the radiation exposure was reduced by 5.5 and 45 times compared to the standard low-dose and conventional radiography respectively. However, details on methodology to measure and calculate the air kerma have not been fully presented as air kerma measures the amount of kinetic energy deposited or absorbed in a unit mass of air which is corresponding to the entrance skin dose [31].

In this study, radiation impact on patients with AIS during whole spine imaging using micro-dose EOS and standard digital radiography (DR) were investigated and compared systematically. Comprehensive measurement of various radiation parameters including entrance skin dose, effective dose and organ dose were included. Entrance skin dose is a direct measurement of radiation output at the point of skin entry for x-ray examinations, and effective dose is a calculated value, commonly in the unit of milli-sivert (mSv) or micro-sivert (μSv), that takes the absorbed dose to all organs of the body, the relative harm level of the radiation and the sensitivities of each organ to radiation into account. Image quality from both techniques was also assessed using criteria for diagnostic radiographic images.

## Methods

The research protocol was approved by the Clinical Research Ethics Committee of the institution and conducted in compliance with the principles of Declaration of Helsinki. Written informed consents were obtained from both volunteers and their parent (or legal guardian). One hundred and thirty-three patients with AIS were recruited from the outpatient clinic and patients with history of scoliosis surgery were excluded. Ninety-nine of them underwent EOS micro-dose protocol, 33 underwent routine digital radiography and one was excluded as EOS standard low-dose was applied eventually. Table 1 shows the demographics of the subjects.

**Table 1:** Demographics of patients

	EOS micro-dose (*n* = 99)	Digital radiography (*n* = 33)	*p*-value
Age	17.9 (4.8)	15.6 (3.5)	0.01*
Gender	18 male, 81 female	11 male, 22 female	
Risser sign	4.2 (1.0)	4.3 (1.0)	0.70
Height (cm)	161.3 (8.3)	161.3 (11.3)	0.96
Weight (kg)	48.6 (6.6)	51.5 (12.6)	0.22
BMI (kg/m^2^)	18.7 (2.2)	19.5 (3.0)	0.10
Cobb angle	31.9 (12.7)	26.3 (12.4)	0.02*

*indicates statistically significant difference at 0.05 level

### Image acquisition

Micro-dose full spine x-ray images were taken from EOS slot-scanning system, newly implemented for radiographic examination, with a total filtration of 0.1 mm copper (Cu) and an x-ray tube anode angle of 7°. Images acquired from digital radiography (Definium 8000, General Electric, United States) with total filtration of 2.7 mm aluminum equivalent employed stitching method to develop a full spine image. All images were taken at PA standing orientation with both arms raised and hands holding the handling bar during the procedure in micro-dose EOS and were protected by collimators in digital radiography.

### Measurement of radiation dose

**Fig. 1:**
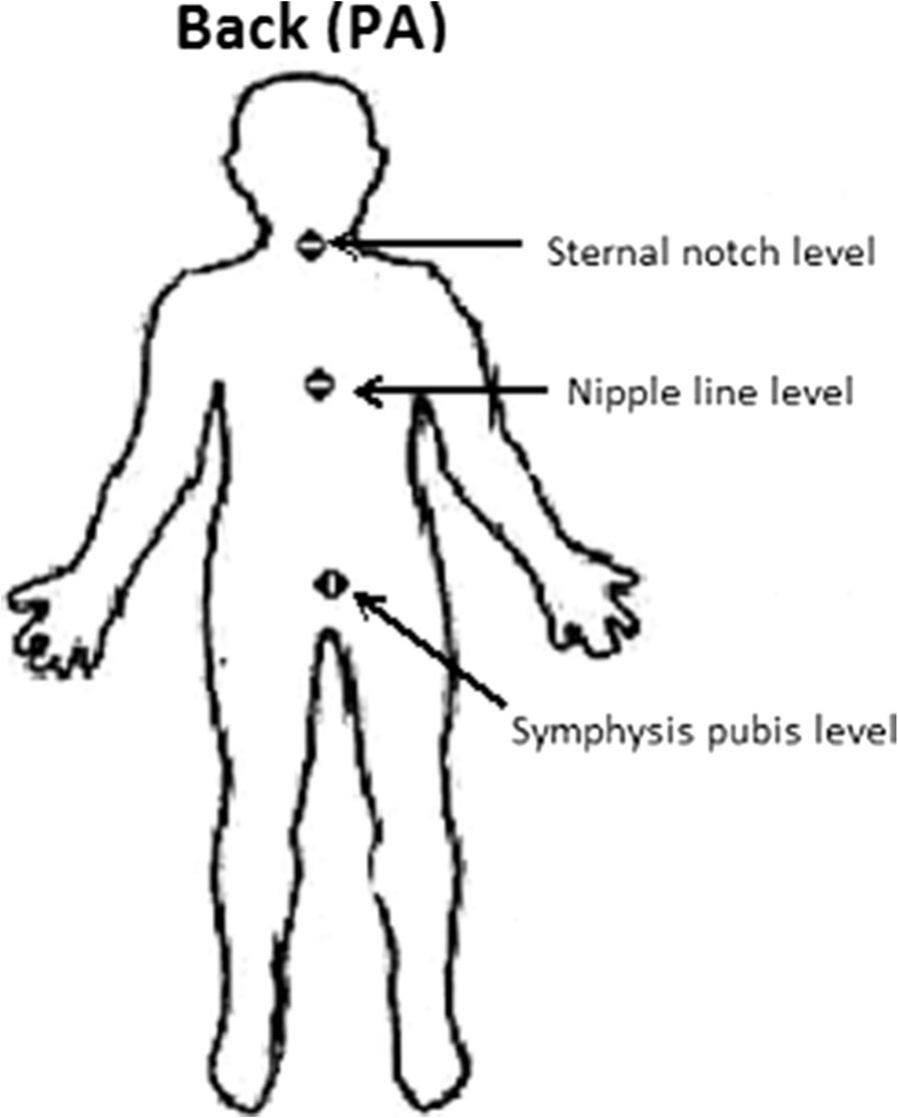
Location of the thermoluminescent dosimeters

All subjects with AIS underwent micro-dose EOS x-ray or digital radiography without brace at PA orientation. Three packs of thermoluminescent dosimeters (TLD-100H) were placed at the back of each subject corresponding to the level of the anterior structures of sternal notch, nipple line and symphysis pubis to measure the level of entrance skin dose as shown in Fig. 1. Irradiated TLD packs were loaded into magazines and readouts were obtained using TLD-Reader (RE-2000, RADOS, Germany). Dose-area product (DAP) was automatically calculated and directly obtained from both EOS system and standard digital radiography.

Effective dose and organ dose were calculated using PCXMC 2.0 [32]. PCXMC 2.0 calculated effective dose as well as organ dose from x-ray examination based on the Monte Carlo method on phantom family from Oak Ridge National Laboratory (ORNL). The simulation required several parameters as shown in Table 2. Focus-to-skin distance (FSD) was the distance between the focal spot of the x-ray tube to the skin of subjects. Other important parameters affected absorbed radiation dose included the area and duration of exposure, input tube current, peak voltage, filters and projection angle.

**Table 2:** Parameters in EOS micro-dose and standard digital radiography

	EOS micro-dose	Digital radiography
FSD (cm)	86.43 (4.56)	163.3 (0.94)
Beam width (cm)	44.22 (1.27)	34.2 (2.51)
Beam height (cm)	76.69 (4.47)	32.9 (3.04) – in 3 sections
Projection angle (degree)	90^o^ (PA)	90^o^ (PA)
X-ray tube potential (kv)	60.7 (1.83)	78.2 (5.9)
X-ray current (mA)	80.8 (3.96)	402.3 (56.0)
X-ray tube anode angle (degree)	7^o^	12^o^
Filter	Copper (Cu)	Aluminum (Al)
Filter thickness (mm)	0.1	2.7
Scanning Time (sec/msec)	7.72 (0.65)	12.09 (3.92) Sternal notch 33.03 (13.72) Nipple line 53.14 (18.04) Symphysis pubis
Monte Carlo simulation parameters: Maximum energy	150 keV	150 keV
Monte Carlo simulation parameters: Number of photons	20000	20000

Standard digital radiography used stitching method to connect three sections of the x-ray into a full spine image. So the simulation in PCXMC was also performed in three sections to calculate the effective dose and organ dose based on DAP and TLD reading at the dorsal sites at the level of sternal notch, nipple line and pubic symphysis level. Scanning range and area of measurement were obtained during the procedure. EOS imaging employed the slot-scanning technique to obtain the spinal images. Continuous scanning allowed one single shot radiation exposure avoiding repeated exposures in duplicated regions, which often happened in standard digital radiography. On each patient, only one simulation, (including the full body), was performed to calculate effective dose and organ dose for EOS micro-dose protocol.

### Evaluation of image quality

Images obtained from EOS micro-dose and standard digital radiography were compared using inter-observer variation based on Cobb angle measurement and image quality evaluation according to Kogon et al [33] and Cook et al [34]. Two raters, who had undergone training to measure Cobb angle using standardized method with over 3 years of experience in AIS related research, performed the rating independently.

### Data analysis

Equality of variances was measured by Levene’s Test and equality of means of DAP, entrance skin dose, effective dose, and organ dose were analyzed between group by independent samples t-test using SPSS 20 (SPSS, Chicago, IL). Results were further divided into gender groups (e.g. female in EOS, male in EOS, female in DR and male in DR) and comparisons between different genders were also conducted by independent samples t-test within EOS and digital radiography. Results were presented in mean and standard deviation and statistical significant level was set at *p* < 0.05. Intra-class correlation coefficient (ICC) was used to measure inter-rater reliability from Cobb angles obtained from two raters. It allowed us to evaluate whether or not image quality from micro-dose x-ray or standard digital radiography would affect raters’ consistency in measuring Cobb angle. Image quality evaluation according to Kogon et al [33] and Cook et al [34] allowed the assessment for optimization of images obtained from micro-dose EOS and standard digital radiography. All nine parameters plus the overall rating were compared between EOS and digital radiography using non-parametric Mann–Whitney U test for ordinal data.

## Results

Significant differences (*p* < 0.001) were obtained in DAP, entrance skin dose, effective dose and organ dose between EOS micro-dose and standard digital radiography as shown in Table 3. Entrance skin dose obtained from the dorsal sites at the level of sternal notch, nipple line and pubic symphysis were 25.0 μGy, 26.0 μGy and 27.2 μGy respectively in EOS and 140.9 μGy, 521.4 μGy and 724.9 μGy in digital radiography. In terms of ratio, they were 5.6, 20.0 and 26.7 times less respectively in EOS compared to standard digital radiography. The effective dose of a full spine PA x-ray was 2.6 μSv in EOS and 67.5 μSv in digital radiography. The organ dose at the thyroid, lung and reproductive organ (e.g. ovaries in female and testes in male) were 0.80 μGy, 5.3 μGy and 2.0 μGy respectively from EOS, and 12.3 μGy, 108.5 μGy and 68 μGy respectively from digital radiography. In terms of ratio, they were 15.4, 20.5 and 34.0 times less in micro-dose EOS.

**Table 3:** Statistical results of radiation dose between EOS and standard digital radiography

	EOS (*n* = 99)	Digital Radiography (*n* = 33)	Ratio (DR/EOS)	*p*-value
Entrance Skin Dose (μGy)				
- Sternal Notch^a^	25.0 (4.8)	140.9 (49.6)	5.6	<0.001*
- Nipple Line^a^	26.0 (4.7)	521.4 (216.4)	20.1	<0.001*
- Symphysis Pubis^a^	27.2 (5.1)	724.9 (295.7)	26.7	<0.001*
Effective Dose (μSv)	2.6 (0.5)	67.5 (23.3)	26.0	<0.001*
Organ Dose (μGy)				
- Thyroid	0.80 (0.3)	12.3 (4.0)	15.5	<0.001*
- Lung	5.3 (1.0)	108.5 (39.7)	20.5	<0.001*
- Reproductive Organ	2.0 (1.0)	68.0 (34.6)	34.5	<0.001*
DAP (mGycm^2^)	39.8 (7.2)	609.5 (263.6)	15.3	<0.001*

* indicates statistically significant difference at 0.05 level

^a^ entrance skin doses were obtained at dorsal sites (the back of each volunteer) at the level of corresponding anterior structures

For results further divided into gender, within group difference was compared using independent t-test. Results indicated that no significant difference was found in effective dose between gender (*p* = 0.35 in EOS, *p* = 0.231 in digital radiography). However, in specific region, organ dose at the reproductive organ (e.g. ovaries in female and testes in male) was significantly higher in female (*p* < 0.001 in both EOS and digital radiography) as shown in Tables 4 and 5. Entrance skin dose at dorsal sites at the level of sternal notch was significantly lower in female in both EOS and standard digital radiography (*p* = 0.023 in EOS, *p* = 0.013 in digital radiography).

**Table 4:** Results from EOS micro-dose in different gender group

	Female in EOS (*n* = 81)	Male in EOS (*n* = 18)	*p*-value
Entrance Skin Dose (μGy)			
- Sternal Notch^a^	24.5 (4.4)	27.2 (5.7)	0.02*
- Nipple Line^a^	25.6 (4.3)	28.0 (5.9)	0.05
- Symphysis Pubis^a^	26.9 (5.1)	28.3 (5.0)	0.33
Effective Dose (μSv)	2.57 (0.48)	2.70 (0.67)	0.35
Organ Dose (μGy)			
- Thyroid	0.79 (0.28)	0.77 (0.25)	0.71
- Lung	5.25 (0.96)	5.53 (1.35)	0.31
- Reproductive Organ	2.29 (0.79)	0.54 (0.20)	<0.01*
DAP (mGycm^2^)	38.8 (6.32)	44.2 (9.30)	0.03*

*indicates statistically significant difference at 0.05 level

^a^entrance skin doses were obtained at dorsal sites (the back of each volunteer) at the level of corresponding anterior structures

**Table 5:** Results from standard digital radiography in different gender group

	Female in DR (*n* = 22)	Male in DR (*n* = 11)	*p*-value
Entrance Skin Dose (μGy)			
- Sternal Notch^a^	126.1 (33.2)	170.5 (64.1)	0.01*
- Nipple Line^a^	469.6 (165.2)	625.1 (273.5)	0.05
- Symphysis Pubis^a^	709.3 (276.4)	756.1 (343.4)	0.68
Effective Dose (μSv)	64.0 (21.1)	74.4 (26.9)	0.23
Organ Dose (μGy)			
- Thyroid	11.6 (3.1)	13.5 (5.3)	0.31
- Lung	99.8 (31.5)	126.1 (49.5)	0.07
- Reproductive Organ	83.6 (31.4)	36.8 (12.9)	<0.01*
DAP (mGycm^2^)	546.9591 (219.2)	734.7273 (309.1)	0.05

*indicates statistically significant difference at 0.05 level

^a^entrance skin doses were obtained at dorsal sites (the back of each volunteer) at the level of corresponding anterior structures

Cobb angles were measured by two raters independently, and ICC indicated that the inter-rater reliability was significantly correlated (*p* < 0.001) in EOS (ICC = 0.883) and standard digital radiography (ICC = 0.942). For the image quality assessment, overall ratings in EOS were 20.4 and 20.1 from rater 1 and rater 2, respectively, and in digital radiography were 20.3 and 19.6 from rater 1 and 2, respectively as shown in Table 6, with higher ratings indicated a better image quality and vice versa. Results from rater 1 indicated that Collimation (*p* = 0.012) and Details (*p* = 0.001) were significantly different between the two modalities. Collimation was better in EOS but Detail was better in digital radiography. The rests were not significantly different. Results from rater 2 were in agreement with rater 1 that Collimation (*p* = 0.010) were better in EOS whereas Details (*p* = 0.001) were significantly better in digital radiography, while rotation was at marginal difference (*p* = 0.065). Details are shown in Table 6.

**Table 6:** Average ratings from Rater 1 and 2 on images from EOS and digital radiography

	Rater 1 EOS	Rater 1 DR	*P* value	Rater 2 EOS	Rater 2 DR	*p*-value
Rotation (0–3)	2.8	2.5	0.15	2.7	2.4	0.07
Tilting (0–3)	2.6	2.4	0.58	2.7	2.6	0.78
Collimation (0–4)	3.0	2.7	0.01*	2.9	2.6	0.01*
Collimation marks seen (0–3)	0.0	0.0	1.00	0.0	0.0	1.00
Presence of holder’s hands (0–3)	3.0	3.0	1.00	3.0	3.0	1.00
Gonad/additional lead protection (0–3)	0.0	0.0	1.00	0.0	0.0	1.00
Artefacts (0–3)	3.0	2.8	0.16	2.8	2.7	0.49
Details (0–3)	2.0	2.8	0.001*	2.2	2.7	0.01*
Density (0–4)	4.0	3.7	0.53	3.7	3.6	0.45
Overall rating	20.2	20.0	0.98	20.1	19.6	0.90

*indicates statistically significant difference at 0.05 level

## Discussion

Entrance skin dose (ESD) was a direct measurement of radiation absorbed by skin. It was measured in the unit of gray (Gy) which one Gy of the dose was equivalent to one joule of energy deposited in a kilogram of matter (J/kg). In this study, it was measured at the back of each subject corresponding to the level of sternal notch, nipple line and pubic symphysis. These three regions were selected due to the relatively high radio-sensitivity of their corresponding tissues/organs (e.g. thyroid, lung, breast, and gonads) [35]. Micro-dose EOS produced consistent air kerma with the slot-scanning technique. ESD exposed on patients was therefore very stable at all three regions at a very low dose as shown in Table 3. In standard digital radiography, ESD varied and the highest dose was measured at pubic symphysis mainly due to the longer exposure time. In both EOS and standard digital radiography, male received significantly higher ESD at dorsal site of sternal notch (*p* = 0.023 in EOS, *p* = 0.013 in DR) compared to female, due to larger input current, longer duration of exposure and a larger area of exposure corresponding to their body size. To further conduct the assessment on biological effect of radiation on patients who underwent x-ray, effective dose was calculated using PCXMC simulation.

All effective doses presented in this study were based on the weighting factor in the latest update from ICRP 103 [35]. Effective dose considered the biological effectiveness of different types of tissues. It was defined as the multiplication of equivalent dose to tissue weighting factor at specific organ. Patients who underwent micro-dose EOS were only exposed to about 3.9% of the effective dose in standard DR from a full spine procedure at PA orientation. The reduction was approximately 26 times (2.6 μSv in EOS versus 67.5 μSv in standard DR). Based on in-house data of 513 patients with AIS from the out-patient clinic, the average follow-up duration was 4.6 years at a 6 month interval starting at the age of 13.5 years old. Estimated number of x-ray taken would be 9.2, which meant patients with AIS in average could reduce almost 600 μSv of effective dose from using micro-dose in accumulation from their adolescent stage or equivalent to approximately one third to a quarter of effective dose from natural background radiation in a year [36]. The effective dose of a single micro-dose x-ray (2.6 μSv) was less than a day of background radiation [36]. Specific organs were taken into account due to their greater radio-sensitivity and organ dose at thyroid, lung and reproductive organs were compared.

In organ dose comparison, data were divided into male and female. Organ dose was the absorbed dose averaged over an organ. Results from EOS and standard digital radiography both indicated that female received significantly higher dose at ovaries compared to testes. It could possibly explain why female patients with AIS had higher risks of unsuccessful attempts at pregnancy, spontaneous abortions and abnormalities in infants as suggested by Goldberg et al. [8]. While the accumulated ionizing radiation could possibly induce adverse effects on both patients underwent x-ray and the development of their fetus in long term, micro-dose protocol became exceptionally valuable to the vulnerable group of adolescent especially in females. The lungs in males was exposed to the greatest organ dose due to its high radio-sensitivity and relatively large volume. But no statistically significant difference was observed within gender groups (*p* = 0.31 in EOS, *p* = 0.07 in DR). Besides radiation dose, image quality was also assessed.

**Fig. 2:**
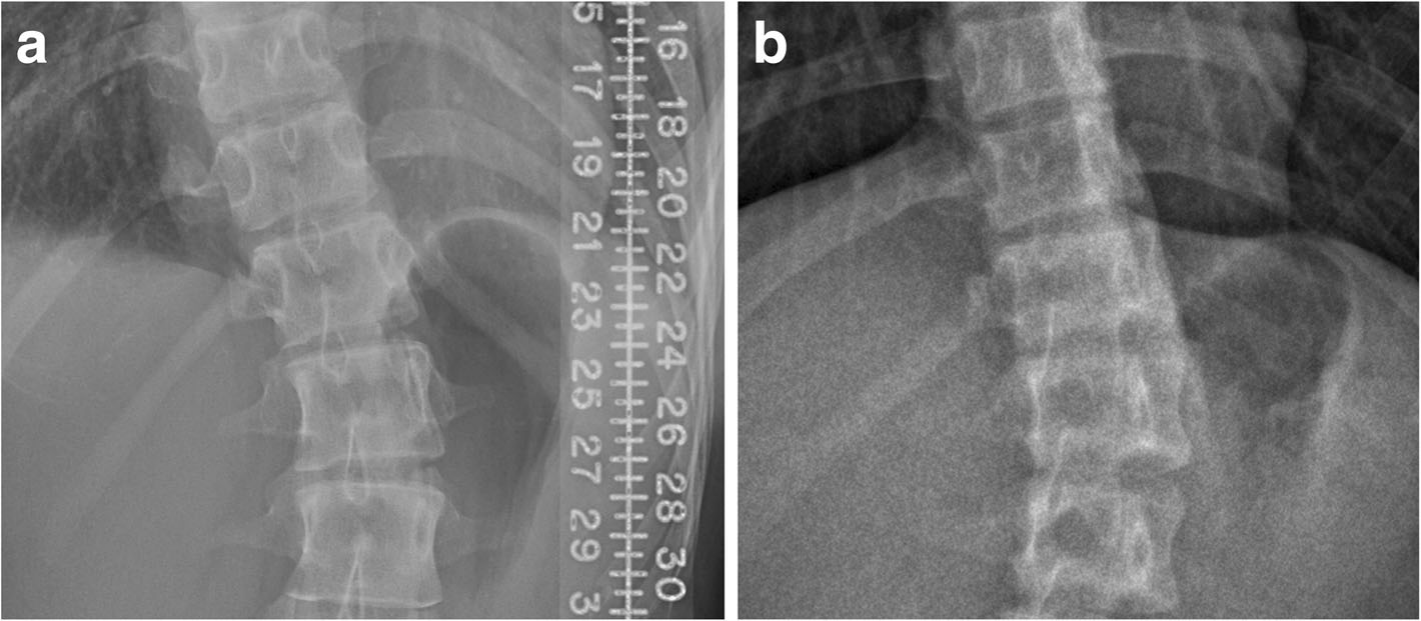
Image comparison between **a** standard digital radiography and **b** micro-dose EOS

Thirty full spine x-ray images were selected from each imaging protocol. Cobb angle was independently measured by two raters who had at least 3 years of research experience in AIS. ICC indicated satisfactory inter-rater reliability, which suggested that images from micro-dose protocol and standard digital radiography did not affect the consistency in angle measurement. Image quality was further assessed based on the rating on nine parameters as shown in Table 6. Both raters rated significantly lower scores on the parameter of Details in micro-dose EOS mainly due to the blurry boundaries at vertebral bodies. In digital radiography, more clear and solid boundaries were observed as shown in Fig. 2. Score in Collimation was statically better in micro-dose EOS due to several reasons, including better positioning of patients during the scan and reduction in presence of undesired body parts. A template was also provided on the platform of the EOS machine to allow patients to stand at a proper position to help centering the regions of interest. Both raters also observed better rotation in micro-dose images, which was also due to better positioning of the patients during the procedure. The template provided on the platform of the EOS machine allowed patients and radiographers to easily adjust a proper position perpendicular to the scanning tube. No significant difference was observed in overall rating between the two protocols. Image assessment suggested images from micro-dose protocol provided enough quality to perform consistent measurement on Cobb angle but might not have high enough resolution to pinpoint fine details for diagnosis, such as for bone metastasis. Considering that patients with AIS received x-ray in routine basis for follow-up purpose, micro-dose x-ray provided images with good enough quality for a physician to evaluate the progression of the curve which was the main objective to undergo x-ray for this group of teenagers. It was worth to reduce the radiation especially in long term accumulation compensated with an acceptable deduction in image details.

Radiation measurement and comparison were conducted on PA plane as the TLD measurement will be difficult to interpret if both PA and lateral views were obtained by the biplanar EOS system due to contamination of the TLD reading by x-ray sources from two different directions. For the patients, age (*p* = 0.01) and Cobb angle (*p* = 0.02) were statistically significant different between the two groups as shown in Table 1. Age could be a potential factor that might affect the effective dose simulation but it was mainly used to estimate the risk of death due to radiation-induced cancer which was not included in this study. There were no significant differences in height (*p* = 0.96) and weight (*p* = 0.22) between the two groups indicated the exposure area would be similar. Also, no significant difference was observed in bone maturity as the Risser sign was similar between groups (*p* = 0.70), indicating an absence of difference in biological response of bone tissues. Cobb angle was not strictly controlled as the main purpose of this study was not investigating the etiology of AIS but to measure the radiation exposure. It was not a parameter for the simulation of effective dose and has no known relation to the radiation, so its effect should be minimal. The unequal sample size would be a concern because majority of the follow up AIS patients underwent micro-dose protocol after the implementation of the EOS machine except for those who could not stand firm and steady underwent digital radiography. The number of AIS patients from digital radiography was limited.

## Conclusions

We concluded that micro-dose EOS provided comparable and clinically useful images of the whole spine for AIS patients while significantly reduced radiation exposure. We suggest that patients with AIS undergo initial x-ray with standard digital radiography to eliminate differential diagnosis and micro-dose EOS for follow-up purpose, given that no suspicion of bone metastasis, fracture or other complaints existed, to reduce the accumulation of ionizing radiation in long term.

## Abbreviations

AIS: Adolescent idiopathic scoliosis
ALARA: As low as reasonably achievable
AP: Anterior-posterior
DAP: Dose-area product
DR: Digital radiography
FSD: Focus-to-skin distance
ICC: Intra-class correlation coefficient
MWPC: Multi-wire proportional chamber
PA: Posterior-anterior
TLD: Thermoluminescent dosimeters

## References

[1] Weinstein SL, Dolan LA, Cheng JC, Danielsson A, Morcuende JA (2008). Adolescent idiopathic scoliosis. Lancet.

[2] Deng M, Hui SC, Yu FW, Lam TP, Qiu Y, Ng BK (2015). MRI-based morphological evidence of spinal cord tethering predicts curve progression in adolescent idiopathic scoliosis. Spine J.

[3] Shah DJ, Sachs RK, Wilson DJ (2012). Radiation-induced cancer: a modern view. Br J Radiol.

[4] Thompson LH (2012). Recognition, signaling, and repair of DNA double-strand breaks produced by ionizing radiation in mammalian cells: the molecular choreography. Mutat Res.

[5] Levy AR, Goldberg MS, Hanley JA, Mayo NE, Poitras B (1994). Projecting the lifetime risk of cancer from exposure to diagnostic ionizing radiation for adolescent idiopathic scoliosis. Health Phys.

[6] Doody MM, Lonstein JE, Stovall M, Hacker DG, Luckyanov N, Land CE (2000). Breast cancer mortality after diagnostic radiography: findings from the U.S. Scoliosis Cohort Study. Spine (Phila Pa 1976).

[7] Bone CM, Hsieh GH (2000). The risk of carcinogenesis from radiographs to pediatric orthopaedic patients. J Pediatr Orthop.

[8] Goldberg MS, Mayo NE, Levy AR, Scott SC, Poitras B (1998). Adverse reproductive outcomes among women exposed to low levels of ionizing radiation from diagnostic radiography for adolescent idiopathic scoliosis. Epidemiology.

[9] Akhtar W, Aslam M, Ali A, Mirza K, Ahmad N (2008). Film retakes in digital and conventional radiography. J Coll Physicians Surg Pak.

[10] Luo TD, Stans AA, Schueler BA, Larson AN (2015). Cumulative Radiation Exposure With EOS Imaging Compared With Standard Spine Radiographs. Spine Deformity.

[11] Levy AR, Goldberg MS, Mayo NE, Hanley JA, Poitras B (1996). Reducing the lifetime risk of cancer from spinal radiographs among people with adolescent idiopathic scoliosis. Spine (Phila Pa 1976).

[12] Knott P, Pappo E, Cameron M, Demauroy J, Rivard C, Kotwicki T (2014). SOSORT 2012 consensus paper: reducing x-ray exposure in pediatric patients with scoliosis. Scoliosis.

[13] Charpak G, Bouclier R, Bressani T, Favier J, Zupancic C (1968). Use of Multiwire Proportional Counters to Select and Localize Charged Particles. Nucl Instrum Methods.

[14] Amzallag-Bellenger E, Uyttenhove F, Nectoux E, Moraux A, Bigot J, Herbaux B (2014). Idiopathic scoliosis in children and adolescents: assessment with a biplanar X-ray device. Insights Imaging.

[15] Begon M, Scherrer SA, Coillard C, Rivard CH, Allard P (2015). Three-dimensional vertebral wedging and pelvic asymmetries in the early stages of adolescent idiopathic scoliosis. Spine J.

[16] Courvoisier A, Drevelle X, Vialle R, Dubousset J, Skalli W (2013). 3D analysis of brace treatment in idiopathic scoliosis. Eur Spine J.

[17] Briot K, Fechtenbaum J, Etcheto A, Kolta S, Feydy A, Roux C (2015). Diagnosis of vertebral fractures using a low-dose biplanar imaging system. Osteoporos Int.

[18] Buck FM, Guggenberger R, Koch PP, Pfirrmann CW (2012). Femoral and tibial torsion measurements with 3D models based on low-dose biplanar radiographs in comparison with standard CT measurements. AJR Am J Roentgenol.

[19] Rosskopf AB, Ramseier LE, Sutter R, Pfirrmann CW, Buck FM (2014). Femoral and tibial torsion measurement in children and adolescents: comparison of 3D models based on low-dose biplanar radiography and low-dose CT. AJR Am J Roentgenol.

[20] Kadoury S, Shen J, Parent S (2014). Global geometric torsion estimation in adolescent idiopathic scoliosis. Med Biol Eng Comput.

[21] Meyrignac O, Moreno R, Baunin C, Vial J, Accadbled F, Sommet A (2015). Low-dose biplanar radiography can be used in children and adolescents to accurately assess femoral and tibial torsion and greatly reduce irradiation. Eur Radiol.

[22] Ilharreborde B, Vidal C, Skalli W, Mazda K (2013). Sagittal alignment of the cervical spine in adolescent idiopathic scoliosis treated by posteromedial translation. Eur Spine J.

[23] Sutter R, Pfirrmann CW, Espinosa N, Buck FM (2013). Three-dimensional hindfoot alignment measurements based on biplanar radiographs: comparison with standard radiographic measurements. Skeletal Radiol.

[24] Morvan G, Mathieu P, Vuillemin V, Guerini H, Bossard P, Zeitoun F (2011). Standardized way for imaging of the sagittal spinal balance. Eur Spine J.

[25] Lazennec JY, Rousseau MA, Rangel A, Gorin M, Belicourt C, Brusson A (2011). Pelvis and total hip arthroplasty acetabular component orientations in sitting and standing positions: measurements reproductibility with EOS imaging system versus conventional radiographies. Orthop Traumatol Surg Res.

[26] Somoskeoy S, Tunyogi-Csapo M, Bogyo C, Illes T (2012). Accuracy and reliability of coronal and sagittal spinal curvature data based on patient-specific three-dimensional models created by the EOS 2D/3D imaging system. Spine J.

[27] Glaser DA, Doan J, Newton PO (2012). Comparison of 3-dimensional spinal reconstruction accuracy: biplanar radiographs with EOS versus computed tomography. Spine (Phila Pa 1976).

[28] Kalifa G, Charpak Y, Maccia C, Fery-Lemonnier E, Bloch J, Boussard JM (1998). Evaluation of a new low-dose digital x-ray device: first dosimetric and clinical results in children. Pediatr Radiol.

[29] Deschenes S, Charron G, Beaudoin G, Labelle H, Dubois J, Miron MC (2010). Diagnostic imaging of spinal deformities: reducing patients radiation dose with a new slot-scanning X-ray imager. Spine (Phila Pa 1976).

[30] Damet J, Fournier P, Monnin P, Sans-Merce M, Ceroni D, Zand T, et al. Occupational and patient exposure as well as image quality for full spine examinations with the EOS imaging system. Med Phys. 2014;41(6):063901. http://onlinelibrary.wiley.com/wol1/doi/10.1118/1.4873333/full. 10.1118/1.4873333 24877841

[31] Ilharreborde B, Ferrero E, Alison M, Mazda K (2016). EOS microdose protocol for the radiological follow-up of adolescent idiopathic scoliosis. Eur Spine J.

[32] Servomaa A, Tapiovaara M (1998). Organ Dose Calculation in Medical X Ray Examinations by the Program PCXMC. Radiat Prot Dosim.

[33] Kogon PL, Lumsden R (1993). How do you critique your radiographs?. J Can Chiropr Assoc.

[34] Cook JV, Kyriou JC, Pettet A, Fitzgerald MC, Shah K, Pablot SM (2001). Key factors in the optimization of paediatric X-ray practice. Br J Radiol.

[35] The 2007 Recommendations of the International Commission on Radiological Protection. ICRP publication 103. Ann ICRP. 2007; 37:1-332. 10.1016/j.icrp.2007.10.003 18082557

[36] Canadian Nuclear Safety Commission. Fact sheet – Natural background radiation. 2014-11-19 2013. http://nuclearsafety.gc.ca/eng/pdfs/Fact_Sheets/Fact-Sheet-Background-Radiation-eng.pdf. Accessed Nov 2014.

